# The Professional Identity of STEM Faculty as Instructors of Course-based Research Experiences

**DOI:** 10.3389/feduc.2024.1442306

**Published:** 2024-10-23

**Authors:** David Hanauer, Richard Alvey, Ping An, Christa Bancroft, Kristen Butela, Kari Clase, Sean Coleman, D. Parks Collins, Stephanie Conant, Pamela Connerly, Bernadette Connors, Megan Dennis, Erin Doyle, Dustin Edwards, Christy Fillman, Ann Findley, Victoria Frost, Maria Gainey, Urszula Golebiewska, Nancy Guild, Sharon Gusky, Allison Johnson, Kristen Johnson, Karen Klyczek, Julia Lee-Soety, Heather Lindberg, Matthew Mastropaolo, Julie Merkle, Jon Mitchell, Sally Molloy, Fernando Nieto-Fernandez, Jillian Nissen, Tiara Perez Morales, Nick Peters, Susanne Pfeifer, Richard Pollenz, Mary Preuss, German Rosas-Acosta, Margaret Saha, Amy Sprenkle, C. Nicole Sunnen, Deborah Tobiason, Sara Tolsma, Vassie Ware, Yesmi Patricia Ahumada-Santos, Regina Alvarez, Justin Anderson, Mary Ayuk, María Elena Báez-Flores, Dondra Bailey, Frederick Baliraine, Elizabeth Behr, Andrea Beyer, Suparna Bhalla, Lisa Bono, Donald Breakwell, Christine Byrum, Iain Duffy, Alyssa Gleich, Melinda Harrison, Renee Ho, Lee Hughes, Jacob Kagey, Kathryn Kohl, Sean McClory, Alison Moyer, María Alejandra Mussi, Holly Nance, Imade Nsa, Shallee Page, Jesus Ricardo Parra-Unda, Jessica Rocheleau, Sarah Swerdlow, Kara Thoemke, Megan Valentine, Quinn Vega, Catherine Ward, Daniel Williams, Ellen Wisner, William Biederman, Steven Cresawn, Mark Graham, Graham Hatfull, Danielle Heller, Deborah Jacobs-Sera, Denise Monti, Pushpa Ramakrishna, Daniel Russell, Viknesh Sivanathan

**Affiliations:** 1 Department of English, Indiana University of Pennsylvania, Indiana, PA; 2 Biology, Illinois Wesleyan University, Bloomington, IL; 3 Department of Biological Sciences, University of Pittsburgh, Pittsburgh, PA; 4 Biological Sciences, University of Southern California, Los Angeles, CA; 5 Agricultural and Biological Engineering, Purdue University , West Lafayette, IN; 6 Biology, Wartburg College, WAVERLY, IA; 7 Natural Sciences, Mitchell Community College, Statesville, NC; 8 Department of Biology, University of Detroit Mercy, Detroit, MI; 9 School of Natural Sciences, Indiana University Southeast, New Albany, IN; 10 Science and Math, Dominican University New York, Orangeburg, NY; 11 Biology, Marist College, Poughkeepsie, NY; 12 Department of Biology, Doane University, Crete, NE; 13 Biological Sciences, Tarleton State University, Stephenville, TX; 14 Molecular, Cellular, and Developmental Biology, University of Colorado Boulder, Boulder, CO; 15 Biology - School of Sciences, University of Louisiana at Monroe, Monroe, LA; 16 Department of Biology, Winthrop University, Rock Hill, SC; 17 Chemistry and Physics, Western Carolina University, Cullowhee, NC; 18 Biological Sciences and Geology, Queensborough Community College, CUNY, Bayside, NY; 19 STEM, Connecticut State Community College: Northwestern, Winsted, CT; 20 Center for Biological Data Science, Virginia Commonwealth University, Richmond, VA; 21 Department of Life Sciences, University of New Hampshire Manchester, Manchester, NH; 22 Biology Department, University of Wisconsin-River Falls, River Falls, WI; 23 Department of Biology, Saint Joseph’s University, Philadelphia, PA; 24 Biology, Virginia Western Community College, Roanoke, VA; 25 Sciences, Neumann University, Aston, PA; 26 Department of Biology, University of Evansville, Evansville, IN; 27 Science and Mathematics, Northern State University, Aberdeen, SD; 28 Molecular and Biomedical Sciences, University of Maine, Orono, ME; 29 Biological Sciences, SUNY Old Westbury, Old Westbury, NY; 30 Biological Sciences, Benedictine University, Lisle, IL; 31 Plant Pathology, Entomology, and Microbiology, Iowa State University, Ames, IA; 32 Center for Evolution and Medicine, School of Life Sciences, Arizona State University, Tempe, AZ; 33 Molecular Biosciences, University of South Florida, Tampa, FL; 34 Natural Sciences and Mathematics, Webster University, Webster Groves, MO; 35 Biological Sciences, The University of Texas at El Paso (UTEP), El Paso, TX; 36 Biology, William and Mary, Williamsburg, VA; 37 Biology, Salem State University, Salem, MA; 38 Biology, Carthage College, Kenosha, WI; 39 Department of Biology, Northwestern College, Orange City, IA; 40 Biological Sciences, Lehigh University, Bethlehem, PA; 41 Unidad de Investigaciones en Salud Pública. Facultad de Ciencias Químico Biológicas., Universidad Autónoma de Sinaloa, Culiacán, Sinaloa., Mexico; 42 Department of Biological Sciences, Southeastern Louisiana University, Hammond, LA; 43 Biology, Howard University, Washington, DC; 44 Department of Natural Sciences, Coppin State University, Baltimore, MD; 45 Biology & Kinesiology, LeTourneau University, LONGVIEW, TX; 46 Biology, Madison Area Technical College, Madison, WI; 47 Department of Biology, Virginia State University, Petersburg, VA; 48 Natural Science, Mount Saint Mary College, Newburgh, NY; 49 Department of Biological Sciences, Texas Tech University, Lubbock, TX; 50 Microbiology and Molecular Biology, Brigham Young University, Provo, UT; 51 Department of Biology, College of Charleston, Charleston, SC; 52 Department of Natural Sciences, Saint Leo University, Saint Leo, FL; 53 Biological Sciences, State University of New York, College at Plattsburgh, Plattsburgh, NY; 54 Science Department, Cabrini University, Radnor, PA; 55 Department of Biological Sciences, University of North Texas, Denton, Texas; 57 Natural Sciences Department, La Salle University, Philadelphia, PA; 58 Department of Biology, Drexel University, Philadelphia, PA; 59 Departamento de Microbiología Básica, Facultad de Ciencias Bioquímicas y Farmacéuticas-UNR, Rosario, Argentina; 60 Natural Sciences, College of Coastal Georgia, Brunswick, GA; 61 Microbiology, University of Lagos, Lagos, Nigeria, Lagos; 62 College of Health and Natural Sciences, Franklin Pierce University, Rindge, NH; 63 Biology, University of Massachusetts, Amherst, Amherst, MA; 64 Biological Sciences, University of Pittsburgh, Greensburg, Greensburg, PA; 65 Biology, The College of St. Scholastica, Duluth, MN; 66 Biological Sciences, SUNY Plattsburgh, Plattsburgh, NY; 67 Biology, Montclair State University, Montclair, NJ; 68 Biological Sciences, Durham Technical Community College, Durham, NC; 69 Biology, Coastal Carolina University, Conway, SC; 70 Biological Sciences, University of North Carolina at Charlotte, Charlotte, NC; 71 Center for the Advancement of Science Leadership and Culture, Howard Hughes Medical Institute, Chevy Chase, MD; 72 Department of Biology, James Madison University, Harrisonburg, VA; 73 Department of Ecology and Evolutionary Biology, Yale University, New Haven, CT

## Abstract

The professional identity of scientists has historically been cultivated to value research over teaching, which can undermine initiatives that aim to reform science education. Course-Based Research Experiences (CRE) and the inclusive Research and Education Communities (iREC) are two successful and impactful reform efforts that integrate research and teaching. The aim of this study is to explicate the professional identity of instructors who implement a CRE within an established iREC and to explore how this identity contributes to the success of these programs. 97 CRE instructors from the Science Education Alliance (SEA) iREC participated in a 2-year, multi-stage, qualitative research project that involved weekly reflective journaling, autoethnographic description, small group evaluation and writing, and large-scale community checking. The resulting description of professional identity consisted of shared *values* (inclusivity, student success, community membership, ownership/agency, science, overcoming failure, and persistence), specified *roles* (mentor, advocate, scientist, educator, motivator, collaborator, community builder, learner, evaluator and project manager) and a stated *sense of self* (dedicated, resilient, pride in students, multiskilled, valued, community member, responsible and overworked). Analysis of individual reflective diary entries revealed how a professional identity underpinned and facilitated the ways in which faculty addressed challenges that arose and worked towards the success of every student. It is the self-concept of the professional identity of the instructor in the context of the CRE classroom that directed the extended commitment and effort that these instructors evidently put into their work with students, which facilitated student engagement, student persistence, and their collective scientific output. The study concludes that a professional identity of STEM faculty in the context of a CRE and iREC combines being a researcher and educator, and that this integrated identity is central for current initiatives aimed at transforming undergraduate STEM education.

## Introduction

Longstanding calls for transformation in undergraduate science education highlight the inertia of change at a national level (PCAST, 2012; Brewer & Smith, 2011). Brownell & Tanner (2012) raise as a key factor undermining transformation efforts in STEM the professional identity of STEM faculty, which has historically been cultivated to value research over teaching and which has created tension between these two roles of STEM faculty. Over the last decade and a half, two broadly adopted, highly impactful and related curricular reform efforts in STEM, namely Course-based Research Experiences (CREs) and the inclusive Research and Education Community (iREC), have integrated research and teaching and may thus operate in a manner that resolves the tension between the two aspects STEM faculty identity, if not synergizes them (Auchincloss et al., 2014; Graham et al., 2013; Hanauer et al., 2017; Hanauer et al., 2022; Rodenbusch et al., 2016; Monti et al., submitted). Here, we explicate the professional identity of STEM faculty who are engaged in a CRE and iREC and explore how that identity shapes the outcomes of these reform efforts.

CREs are designed to scale up access for undergraduate students to hands-on research experiences that promote their learning and persistence in STEM. By placing research in typical undergraduate laboratory courses (e.g., introductory biology laboratory courses), CREs enable students to readily access authentic research experiences. Over a decade of assessments have made clear that through a CRE, students develop a sense of ownership over their research, agency in their learning, and science identity that together increases their desire and likelihood to remain in the sciences (Russell *et al.*, 2007; Jordan *et al.*, 2014; Hanauer *et al.*, 2017; Hanauer et al., 2022a; Hernandez *et al.*, 2018). Importantly, these outcomes are observed for students from a wide range of demographics. Recent studies exploring the ways in which these student outcomes emerge highlight a central role of CRE pedagogy. In particular, there is a rich suite of conceptual and emotional facilitation by the instructor ranging from modeling scientific thinking to promoting independence and perseverance, all of which takes place across multiple levels, from large group settings to small peer groups and individual one-on-one engagement (Hanauer et al., 2022b). There is also extensive usage of ad-hoc, informal, ungraded formative assessment that is facilitative of student learning (Hanauer et al., 2023). These studies make clear that in a CRE, STEM faculty are functioning in ways that go beyond the usual instructional practices in traditional undergraduate laboratory courses to also assume the role of mentors more typical in academic research laboratory settings.

iRECs were developed to support broad adoption of CRE teaching by STEM faculty. Through an iREC, STEM faculty are provided both pedagogical and research support to lead a CRE and are coordinated as a community that convenes regularly to share and advance their research and pedagogy (Monti et al., submitted). The Science Education Alliance (SEA) by the Howard Hughes Medical Institute (HHMI), Genomics Education Partnership, and Tiny Earth are three well-established iRECs that each support large communities of STEM faculty at dozens or hundreds of colleges and universities (Jordan et al., 2014; Hanauer et al., 2017; Schaffer et al., 2010; Elgin et al., 2017; Hurley et al., 2021).

For these STEM faculty who lead a CRE and supported to do so within the context of an iREC, research and teaching are integrated into a single entity, both within and beyond their classroom (Auchincloss, et al, 2014; Hanauer et al., 2017). Accordingly, the professional identity of these STEM faculty may involve interesting developments in terms of how they see their roles as researchers and educators, which can shape their motivation and pedagogy, the resulting educational experience and outcomes for their students, as well as the overall success of CRE and iREC as STEM reform efforts. To better understand this positioning, the current study aims to explicate the professional identity of these undergraduate STEM faculty in the context of their CRE classroom and explore the relationship between this identity and student outcomes.

## What is Professional Identity?

Social theory has proposed that people that are together in a social setting develop a shared discourse which defines how they experience themselves (Bourdieu, 1993; Gee, 1996). Through conscious and unconscious social processes this shared discourse integrates, among other components, a series of values, beliefs, roles, aims and social understandings into an identity which constructs the individual’s positioning in that social group (Hodkinson &Hodkinson, 2004; LunDell & Collins, 2001). On its simplest level, the professional identity of an instructor relates to a collective set of norms, beliefs, roles and practices which a social grouping within a higher education setting has developed (Beijaard, Meijer, & Verloop, 2004; Kogan, 2000; Kuh & Whitt, 1986; Rhoades, 2007). For college level instructors, these collective identities may have emerged from prior education, exposure to disciplinary discourse, shared experiences within an institution, department or program and explicit position statements for any or all these groups (Kuh & Whitt, 1986; Mendoza, 2007). From all these different sources, professional identities may overlap and are subject to change and development over time (Rhoades, 2007). These identities may also come into conflict with one another forcing the instructor to navigate between different ways of self-positioning (Ibarra, 2007).

Prior research has examined various aspects of instructors’ professional identities. These include investigations into how instructors understand their professional commitments and responsibilities (Nadelson et al, 2015; Wallenburg et al., 2016; Shahabi et al., 2020), the aims of their profession and discipline (D’Arcy, et al, 2019; Hancock & Walsh, 2016), the experiences and ways of being in their professional roles (Cameron et al., 2020; O’Leary & Cantilon, 2020; Wald et al., 2019), and the values associated with their work (D’Arcy, et al, 2019; Hancock & Walsh, 2016). Overall, as argued by Raste & Murthy (2024) a teacher’s professional identity reflects the way a person perceives themselves within the educational setting within which they are working. This professional identity has direct ramifications on the way the person functions and interacts in the pedagogical work and the outcomes for themselves and their students. A professional identity addresses the beliefs, values, roles, commitments and aims of a teacher in a particular educational setting (Beijaard, Meijer, & Verloop, 2004).

Research has also shown that the professional identities of science teachers is different from teachers of other disciplines, and that this identity can differ based on various social contextual factors such as institution, science discipline, program and type of employment, and the degree of support and collegial interaction they experience (Feser & Haak, 2023; Rodrigues & Mogarro, 2019). Reflecting Lave and Wenger’s (1991) situated learning theory and Wenger’s (1998) communities of practice (CoP) model, Chen and Mensah (2022) considered the way in which science teachers integrate aspects of the cultural practices of the communities they belong to into their professional identities. Values, roles, educational attitudes, and behaviors concerning science teachers’ professional identities are acquired and learnt within professional contexts (Pérez Gracia et al., 2019; Shwartz & Dori, 2020). Basically, as predicted by both Lave and Wenger’s’ (1991) and Wenger’s’ (1998) theoretical position on how personal development occurs, participation in an active community of practice facilitates instructor professional identity construction without explicit instruction taking place; it is the participation in the community which directs these identity developments.

In the current study, all STEM faculty who participated were drawn from the SEA iREC and implement the Phage Hunters Advancing Genomics and Evolutionary Science (SEA-PHAGES) CRE project (Hanauer et al., 2017). The iREC has a particular series of features that are important in relation to the developmental aspects of a shared professional identity (Monti et al., submitted). First, an iREC has a well-defined mission of supporting STEM faculty to provide each student with a high-quality educational research experience as means to promote their learning and the persistence, *en masse* and across diverse student demographics (Hanauer et al., 2017). Second, the iREC structure and curriculum provides instructors with recurring opportunities to leverage one another to advance their science and pedagogy. This is accomplished through a centralized scientific and administrative structure that facilitates extensive and continual interactions over the long-term between faculty who are situated in multiple institutions across the US (Hanauer et al., 2017). With the program’s underpinning CRE curriculum being similar for all faculty, there is clear basis for conversation and collaboration in relation to their research and teaching. In line with theories of identity development within Communities of Practice (Wenger, 1998), shared mission, participation, identification, extended engagement with other members of the community should be conducive to the development of a shared professional identity.

## Research Questions

As reviewed above, the professional identity of an instructor has a role in how they conduct their pedagogical work. To date, there has not been a study of the professional identity of instructors who lead CREs. Based on prior research there is reason to believe that instructors have a particular professional identity in a CRE setting that informs and directs their pedagogical practice. The current study aims to explicate the professional identity of instructors in a CRE classroom and explore how this may facilitate positive educational outcomes for their students. The current study was directed by two research questions:

1. What characterizes the professional identity of instructors in the context of a CRE?
2. What is the relationship between the professional identity of a CRE instructor, their pedagogy, and student outcomes?

## Methodology

### Overall Design and Research Stages:

The investigation of professional identity and the way this interacts with inclusive STEM education is complex. In order to avoid oversimplifying aspects of professional identity and the ways in which this identity might interact with the CRE instructors pedagogical work, a 2-year, multi-stage, qualitative research methodology involving a large number of CRE instructors was conducted. The data collection process integrated weekly reflective journaling, autoethnographic description, small group evaluation and writing, and large-scale community checking. Table 1 presents the stages of the project, the process of data collection and the data that is elicited at each stage.

### Participants:

The participants for this study consisted of 97 CRE instructors drawn from the HHMI SEA program. Table 2 summarizes the demographic data collected from the CRE instructors who participated in this study. As can be seen in the table, the majority of respondents were women, were faculty who had tenure, and were fauclty with full-time positions at the Associate or Full Professor levels. However, there was also representation of non-tenured faculty with other rankings. Ethnographic data was not collected from participating faculty. All data was collected according to the ethical guidelines of Indiana University of Pennsylvania, IRB #21-107.

### Instruments:

This study involved multiple stages of data collection. Each stage involved its own instructions. The full prompts for the following stages can be found in Appendix A: **A**. Weekly Reflective Journal Prompt; **B**. Autoethnographic Writing Workshop Prompts; **C.** Small Group Evaluation and Writing Prompts; **D**. Individual Identity Differentiation Writing Prompts; **E**. Community Checking and Validation Prompts following Presented Analysis; & **F.** Community Explication of the Manifestation of Inclusive Education in CRE Instruction

### Procedure:

The process of data collection for this project lasted for two years. The first year of work was conducted by the faculty individually in which they completed a weekly reflection on their teaching in a CRE. The individual entries were not monitored. However, every month a short survey was sent to faculty asking about the number of weeks of entries had been completed. Following the first year of reflective journal writing, two workshops were held with faculty. The first of these workshops directed faculty to read their complete reflective journals, choose 3 items and write a position on their understanding of their professional identity. The second workshop involved faculty hearing each other’s experiences in a small group format and defining a shared definition of professional identity and ways in which each person differed from this shared identity. The list of three chosen journal entries, the shared definition of professional identity and the individual divergence from that shared identity were all submitted as data to the lead researcher of this project. The data was analyzed and modeled in order to see the shared components of a professional identity. The resultant analyses and models were presented to a large group of faculty (n=100 +) in order to check the validity of the analyses, categories and models. This member checking process was held at an annual faculty symposium. Faculty considered each of the components of the analysis in a small group format and then following the symposium submitted their agreements, changes and modifications in an online survey. The final process of data collection involved presenting entries from the journal study which related to the concept of inclusive education to the faculty member for their explication and interpretation. In small groups CRE instructors discuss the entries and following the session each instructor completed a survey providing their interpretation of how inclusive education is manifest in CRE pedagogy.

### Analysis:

As a result of the nature of the data which involved year-long reflective diary entries and considerations of personal and professional identity, a process of analysis which directly involved faculty was utilized. This investigation combined collective autoethnographic approaches (Chang, Ngunjiri & Hernandez, 2013) in which participants are also analysts of their own data with more traditional content analysis approaches (Krippendorf, 1980) to resultant written products. Through prompted individual reflection and small group discussion, faculty chose and defined their own and the shared definition of professional identity. Small group discussion between 3 or 4 faculty involved presenting and debating the features of professional identity. Group discussion was designed to allow a provisional shared and agreed upon view of professional identity to emerge. As described below a second stage of validation of these definitions of professional identity was conducted with a large group of faculty. These processes of analysis and writing produced 3 different written products: a list of 3 explicated experiences from their reflective journals, a statement from each small group on the definition of their shared professional identities and a written statement of their personal identities as CRE instructors.

The submitted written data was analyzed using a content analysis approach. Initially the shared descriptions of professional identity were read by two applied linguistic researchers and a list of general categories for the specification of a shared professional identity were proposed. This process specified three general categories of utterances: 1) The *roles* a CRE instructor may take on; 2) The values a CRE instructor believes in; & 3) The sense of self a CRE instructor may have. Following the specification of these general categories, the specific subcategories of the faculty utterances were defined. For each of these three, a list of specific roles, values and sense of self were defined.

In order to check the specified general categories and subcategories, the full set of explicated reflective journal experiences was read for additional specification of the roles, values and sense of self of professional identity. The aim was to understand the source and the experiences which underpinned each of the categories and subcategories of professional identity. If a specified sub-category could not be substantiated with at least one described experience, it was removed from the list of subcategories. If a role, value or sense of self that had not been specified in the general statements of professional identity but was found to be present in at least three specific experiences it was added to the lists of subcategories. A list of general categories, subcategories, and an overall model of professional identity was defined. The full dataset of reflective data entries was specified according to the model and exemplars which manifest the features of the category and subcategory were chosen through discussion

The results of the content analysis approach were presented to a large group of instructors and each member provided feedback. This feedback consisted of 1) specification of agreement or disagreement with each category and subcategory; 2) proposals for changes and modifications; and 3) new items that needed to be added. The degree of agreement with the model was calculated as a simple percentage of participants stating agreement and proposed modifications and additional were evaluated.

The final analysis related to the explication of the ways in which inclusive education is manifest in CRE instruction. The full set of journal entries was reviewed and entries were categorized according to types of inclusive educational action. All entries were read but not all entries were found to be relevant for the theme of inclusive education. Once the entries had been categorized according to different aspects of inclusive education, representative entries that most accurately represented the theme and offered the desired degree of detail were chosen. In some cases, two vignettes were considered for each theme. These were then discussed by three of the researchers. After this consultation, the faculty who wrote each of the entries were contacted to see if the interpretation was accurate and their willingness to highlight their specific diary contributions in the manuscript. As a final stage all the vignettes were presented all the faculty for their feedback. Once each instructor had submitted their interpretation of the journal entry vignettes, they were read and shared understandings summarized in relation to each vignette.

## Results

### Categories of Professional Identity

To establish general categories of professional identity, an initial analysis was conducted on the small-group, shared descriptions of professional identity. Table 3 presents the results of this analysis. The three general categories of values, roles and sense-of-self were defined in order to organize and analyze the written descriptions provided by the instructors in this study. While this general analytic frame emerged from the reading of the data, there is a rationale based on prior literature that underpins this approach. An analysis of the specific values involved in the professional identity of an instructor in the context of their CRE classroom provides insight into the beliefs that support the actions and decisions taken by each instructor in their pedagogical work. The shared values of a professional specify the aims and goals of these instructor and what they are trying to achieve. The list of roles specified in the small group discussions clarifies the functions and positions that these instructors utilize in order to complete their functions and aims as specified by the values they believe in. The last general category – sense of self – specifies the personal characteristics that instructors feel are part of their professional identities in the context of their CRE classroom.

### Instructor Values

Instructor values refer to ways of behaving that a person considers important in their professional life. Table 4 presents the list of values specified by faculty in the descriptions of professional identity that were validated in their journal entries. Across the full set of values, a specific educational orientation emerges. These instructors are placing an emphasis on the success of all students as members of the scientific community. To achieve these aims, instructors value promoting persistence, understanding and participation in the process of science, and ownership, agency and belonging in the community. These values highlight the importance faculty are placing on creating a positive and supportive educational environment. This suggests that faculty are prioritizing the success students, reflecting a deep commitment to their roles as educators and mentors. This focus on student success is a core component of their professional identity, highlighting their dedication to fostering an inclusive and supportive learning environment. This reinforces the sense that their role includes guiding students through challenges and further fostering a sense of ownership, enthusiasm and belonging in the scientific community. In the community checking process of the general category and the specific items on the values list, 91.43% of faculty agreed with these definitions and items.

### Instructor Roles

Instructor roles refer to the various types of professional functions an instructor has, with all associated expectations and meanings. Table 5 presents the list of roles specified by the CRE instructors in this study. As can be seen these instructors see themselves as performing and providing a range of roles that together support and facilitate student learning, success and persistence within a CRE. Roles such as mentor, advocate, and motivator relate to the interpersonal interaction with the student to facilitate persistence. As seen in prior studies concerning how CRE instruction is conducted, these roles are crucial for helping students stay engaged through the challenges of authentic research. The roles of scientist, learner, collaborator, community builder and project manager are tied directly to the role of a lead researcher (i.e. a lab head). Faculty are working with students on novel projects within the setting of a broader community of scientists who are all contributing to advancing science. As is typical in authentic research, the relations between the instructor and student are flattened in relation to some aspects of the work, with the faculty member and students learning together as the project develops. Finally, the roles of educator and evaluator are the more classic aspects of a teacher’s role and remain a part of CRE teaching. Collectively, this set of roles shows the full gamut of what CRE teaching can involve, spanning those typical of both teachers and researchers. It is important to note that the range of roles that are included in the professional identity of a CRE instructor includes traditional roles such as being a mentor, educator and evaluator, psychosocial responsibilities such as counselor and motivator and the roles of a working scientist such as collaborator, learner, project manager and community builder. This combination of roles reflects the complexity of the what teaching a CRE involves, which goes beyond the traditional concept of teaching.

### Instructor Sense of Self

Instructor sense of self refers to the personal qualities that instructors find relevant for their professional identity as an instructor in the context of their CRE classroom. The full list of items is presented in Table 6. The specific items reflect the challenges and opportunities that these instructors face in their professional work, such as adapting to diverse learners, managing multiple tasks, coping with stress, and collaborating with others. Importantly, this list indicates a set of attitudes and behaviors that are needed to support students in the uncertainties of engaging in authentic research, within a larger community of scientists. The need to be committed, flexible, creative, confident, and responsible underpins the qualities needed by instructors in order to function within the framework of a CRE and iREC. This sense of self is, in turn, leads to a sense of pride and self-value as an instructor, scientist and community member. It can also lead to the feeling of being overworked and overwhelmed with the cognitive, emotional and administrative load of courses of this type. Finally, it is interesting to note that in order to be able to function according to the roles and values of this professional identity, faculty see themselves as multi-skilled, resilient and flexible. Together this set of self-concepts can be seen as a response to actually performing the values and roles that characterize the professional identity of CRE instructors.

### CRE Instructor Professional Identity, Pedagogy, and Student Outcomes

As previously described, instructors in a CRE are functioning in ways that go beyond the usual instructional practices in traditional undergraduate laboratory courses to include the role of mentors more typical in academic research laboratory settings. In particular, instructors provide extensive conceptual and emotional support and ad hoc, informal feedback to their students in a CRE (Hanauer et al., 2022b, Hanauer et al., 2023). With the assumption that professional identity underpins the actions and decisions taken by instructors in their classrooms, then the shared values, roles, and sense of self for instructors in the context of a CRE that is revealed in this study offer a basis for understanding this rich pedagogy. If an instructor sees their role as a scientist, mentor and an educator, then their pedagogy will involve the types of facilitation that nurtures an appreciation for the rigor of science alongside the development of a passion for science. If their value system promotes inclusivity, belonging and student success, then they are going to act to promote engagement and learning for every student. If they see themselves to be dedicated, resilient, valued and multiskilled, they are going to go beyond the usual parameters of what is expected for instruction in typical undergraduate STEM education.

These values, roles, and sense of self for instructors in the context of their CREs may also underlie the equitable outcomes observed for students across diverse demographics (Hanauer et al., 2017, Hanauer et al., 2022a). To explore the ways in which this professional identity manifest as inclusive education, that is actions and pedagogy by the instructor that promotes the success of *all* students engaged in a CRE, the full set of weekly reflections were analyzed by groups of instructors to exemplify diverse ways in which they were manifesting inclusive education in their CRE classroom. Four examples are presented below as vignettes from reflective diary entries.

#### Vignette 1: Accommodating Disability

“I found out today that I have a student who is deaf and will require special accommodations (interpreters and note-taker). I know I can do this in the lecture, but I am 100% sure I cannot do this in the SEA-PHAGES lab. We are masked--how do I deal with this? How do I have interpreters on the Zoom screen and still let the student do the work independently? How do I ensure she is safe? How much can her lab partners participate in this journey? How do I manage the lab to be sure she doesn’t stand out from the group? I called her advisor and special services to figure out who put her in this class. As I think about it, I am excited. Maybe this is the spark she needs to figure out her passion. This is my job to help her use this experience to do just that!

##### Thoughts:

At first, I found myself angry that the student was taking the course because she isn’t a biology major. This is a lot of work to set up the lab. It is my job is to ensure equal access to science and these emotions are not in line with what you believe or are charged with. You state that you fully believe in equity and inclusion, and yet you feel this way. You better dig deep and find a way to make it work for the student, no matter the “amount of work” it takes on your part. You believe in the outcome, and you believe in education. You know this experience is good for the bio majors, so why not for her? Dig deep. It all worked out in the end- she passed, she persisted- she didn’t choose biology as a major, but it helped her FEEL successful as a student. She told her advisor she liked the class and me (was I worried she wouldn’t like me?). I know that I grew as an educator because of this experience.”

This brief reflective diary entry provides an example of how inclusive education is put into practice by the instructor and how professional identity plays a role in this thought process. The presence of a deaf student in the lab raises serious concerns for the instructor. These include an increased workload, worries about the student’s safety, and changes to the core design of authentic research practices like collaboration with peers. But the professional identity of the instructor in the context of their CRE classroom includes the belief in the importance of having a student find a “passion” for science and that the authentic research experience is the way to achieve this. Furthermore, clearly this instructor sees this as part of their position. The instructor connects this to the ideas and beliefs in inclusive education which then translated into a set of behaviors, supports and practices which allowed this student to be successful (and enjoyably) complete her research experience. In this reflective entry, inclusive education emerges from the professional identity of the instructor in the context of their CRE classroom, with the values, roles and sense of self that this entails.

#### Vignette 2: Overcoming Educational Discrepancies

“I was once again surprised at how little my students understood about the nature of science at the beginning of the semester even though I had taught this many times before. But I knew I needed to really empathize with them and get into their frame of mind in order to teach effectively. The specific experience that triggered this feeling was how students said that they never saw a single piece of equipment in the room before and, even after doing the reading, had no idea about the process of science or research. One student asked “is this a science lab? Is this what they look like?” Our program takes only students from underserved backgrounds so this made me feel that we do have a very critical role in making these students feel like they belong - and that this is an uphill struggle sometimes. I re-felt this very strong feeling about the level of knowledge of these students (and how unfair our educational system is that would give some students strong backgrounds and other students virtually nothing). Even though my story was about one student, this was not a single experience – it was 15 students all expressing doubt and asking questions and being nervous about everything they saw in a “science Lab” and wondering how I was going to make them feel like they belonged in this environment. My emotion was “how am I going to get all 15 students from underserved backgrounds to become truly engaged in this process and understand and love what they are doing.” But given that we successfully completed the year and now I know that half the class wanted to be a TA the following year so I now know it turned out OK. But still recall how daunting the feeling was – that I would never be able to engage them the way I wanted. I still feel this sense of “how do I make them love this and develop identity as scientists?” at the beginning of every year.”

In this excerpt, the instructor reflects on the challenge of working with students who have very little if any of the expected scientific knowledge and experience prerequisites to complete a lab course. As seen in the excerpt very basic assumptions like knowing what a lab looks like were absent for some students who came into the instructor’s CRE classroom. As expressed here the professional identity of the instructor includes values of student success, persistence, the role of building community and the deep desire to help these students to become scientists. Importantly the faculty member sees the lab setting within the broader construct of the way society constructs educational inequities and considers their classroom the setting which could help reverse such inequities. This aim goes beyond just addressing scientific knowledge and includes a sense of belonging and even love for science and research. The professional identity of the faculty member as a scientist and as an educator within a research community creates a conceptual framework that sees the educational backgrounds of the students not as a deficit and a precursor for failure, but rather as a significant societal task that needs to be and can be addressed through an authentic research experience.

#### Vignette 3: Overcoming Failure

“Approximately Week #3 into the Phage Discovery portion of the course, folks were having success in getting phage doing serial dilutions, picking a plaque…but one student was only getting empty plates. Just nothing. She was very frustrated. “Failure, Failure, Failure’. However, she kept trying, not saying too much, coming in on off-times, both weekends of the semester…nothing. I would check her plates ahead of time. …I even was doing isolation alongside and I would get plaques, but not her. Crappy sample? We even went back to the sample bag and we even collected different samples. Still nothing. All the time…nothing…but her face was hard to look at. I could see her frustration; especially with her classmates moving forward in the isolation process. I felt terrible for her. She was not having a good experience, first semester in school, first in her family to go to college. I felt her pain and would often take her pain home with me, trying to develop a Plan B, Plan C. Plan D…to get her virus. One day, at the beginning of Week #4. … I checked her plates and saw phage!! LOTs of them. It was also on *Gordonia* instead of the host most of the class was working with. I was so excited and simply could not wait until she came in to check her plates. She came in, went to the incubator, held her plates to the window as usual…expecting nothing again…. I watched her. She stood at the window longer than usual. She glanced around to I guess see if somebody was watching….then got her phone out to snap some photos. Then she came over to show me. She was positively glowing!! “It worked’!! She said ‘Finally, I have something positive to write in my lab data book”. She then showed me the ones she wanted to pick and isolate further. I left her alone and about 1 minutes later, I came back to see how she was doing….she hadn’t started. She was busy sending her mother the photo of her plaques. Apparently her mother had been asking, not understanding about phages or anything science, but she knew her daughter was worried about not getting virus and kept asking her how things were going. The student then asked if she could snap a selfie picture of the two of us with her plate of phages. Never had that happen before, but it was just the best! I felt relieved. Also, most happy. Together, we had made a wonderful day! Reflecting on that….I think the one-on-one attention, extra time off-class time and on the weekends…listening to Spotify while working in the lab, me showing her alongside helped. These are the times I enjoy being a teacher. Showing students one-on-one…it is how I learned and I was able to pass that on”

In this excerpt, the instructor presents a case of working as a mentor with one student who was struggling with her failure to find a phage and have a positive result to report. Several different actions were taken by the faculty member including devising different ways of achieving a positive result, mentoring the student one-on-one, working weekends and out of class with this student. What would motivate an instructor to go to these lengths to support student success? The instructor’s professional identity and her core sense of dedication helps her to go way beyond usual lab practices. This instructor was working side by side with this student allowing her to continue and get past failure. The reward for the student in terms of a sense of ownership, confidence and just joy at science is evident in the description. What could have been an experience that led to the decision to leave STEM, was transformed into a moment of deep pride that needs to be shared with her family. The sense of closeness between the student and faculty member is clear and the value the instructor gets from this experience reinforces her dedication to her profession and why she instructs a CRE.

#### Vignette 4: Persistence through Authentic Science

“My third entry occurred in the middle of the second semester of bioinformatics. I have all the same students from the first semester plus a few more from a different section. This entry was about playing the long game with students, meaning the things we do to support students daily often don’t come to fruition until much later. On this day I was assessing student annotations over spring break. Every single student in my class had turned in annotations for all of their assigned genes. This was a remarkable moment that I celebrated because two of these students had had major difficulties in the first semester. They had plagiarized their final paper in the first semester, were dishonest at times, skipped classes and were overall unengaged. These students were also struggling in their other classes and were on university probation. But on that day, these students had completed their work on time and handed in high quality annotations with notes that demonstrated maximum effort. They hadn’t missed any classes and when I reviewed attendance records at our daily mentoring program (for Phage students) they each had attended at least once weekly. They had also written in their learning journal how much they valued the support in [the daily mentoring program] when they felt intimidated on an assignment. These two students went on to play major roles in the writing of two of the MRA papers that we published this summer. I know that you cannot recruit every student to the mission, but it is worth it to continue to give students what they need and not necessarily what they deserve, even when they make very big mistakes because they just might have the capacity to make the changes they need for successful learning when given the chance.”

What is remarkable in this excerpt from the reflective journal of an instructor is the transition of a pair of students from university probation for scholarly misconduct to published scientists contributing to the research community. The instructor speaks of the “long game” and believing in the value of supporting students. This transformation is situated in the nature of the CRE which directs students to produce scientific knowledge. There are several components of professional identity that play a role here. Being a scientist combined with the understanding that students need to be mentored, motivated and supported with the aim of allowing science to emerge allowed the forbearance in relation to earlier transgressions of the students. The students responded to the authenticity of the research by changing their behavior and really doing the science. It is unclear from the description exactly which practices supported this transformation, but what the instructor’s professional identity facilitated is resilience and a clear understanding of what being a scientist involves.

## Discussion

The aim of this study was to explicate the professional identity of STEM faculty who instruct a CRE through an iREC and to consider the ways in which this identity contributes to the outcomes of these education reform efforts. The collective identity of instructors in the SEA program, as explicated in this study, consists of values related to scientific achievement and student success, which are facilitated by roles that include being a scientist, a mentor and an advocate, and a sense of self that allows for commitment and flexibility in working towards the success of every student. These features of the professional identity of STEM faculty in the context of their CRE classroom clearly explain the rich suite of facilitation that characterize CRE instruction and consequently the positive outcomes observed for students across diverse demographics (Hanauer et al., 2017, Hanauer at al., 2022a, Hanauer et al., 2023).

The interconnectedness of instructor identity, their pedagogy, and student outcomes is exemplified in the analysis of the reflective diary vignettes presented here. In each vignette, the instructor is faced with a challenge and a series of decisions that need to be made. The positionality of the instructor, conceptualized through professional identity, is the intervening factor that directs the way the faculty member acts. In one case, the instructor makes all relevant accommodations to support a deaf student, in another the instructor works one-on-one on weekends and after class to help a student to discover a bacteriophage, and in another case the instructor “plays the long game” and transforms students who are failing and under university probation into publishing scientists contributing to science. It is the professional identity of the instructor in the context of their CRE classroom that drives the extended commitment and effort that these instructors evidently put into their work with students. The outcome of this increased effort is an educational experience that is rich for all students, and which also contributes quality data that advances science.

We infer that the professional identity of STEM faculty described in this study is fostered by particular features of a CRE and iREC. In a CRE, the centrality of authentic research positions faculty and students as researchers working together towards a shared goal of producing scientific outputs of value for the broader community. Consequently, in the role of the experienced scientist, instructors go beyond quality instruction and to also serve as a research mentor who works with each student towards their collective success. These aspects of professional identity are further reinforced through the iREC, which is designed to support each instructor in their role as a researcher and a research instructor and which affords them multiple opportunities to meet and collaborate with other members who share the goals and mission of the broader iREC community (Monti et al., submitted). Features of a CRE and iREC, which foster the professional identity of STEM faculty described in this study, therefore underpin the success of the SEA program and CRE instruction more broadly. \

The analysis presented here, at least as it relates to CRE instructors with the SEA program, also offers a perspective into the tension between being a researcher and a teacher in STEM that was described by Brownell and Tanner (2012). As uncovered in this current study, teaching, per se, is not undervalued by STEM faculty in relation to research. Rather, STEM faculty value teaching when it resembles how learning and development occur for those in the profession; that is, when scientific content, concepts, and skills are taught and learned in the context of engaging in and advancing authentic research. This is true for STEM faculty in the context of graduate education and, as described here, can also be true in undergraduate science education (Feldon et al., 2015). We therefore postulate that STEM faculty have historically undervalued teaching in undergraduate science education, in part, because the undergraduate science curriculum does not reflect how learning and development occur for those in the profession and because it does not contribute towards advancing science. When there is synergy between teaching and research, as in graduate education or a CRE, faculty are evidently committed teachers. Indeed, as described in Monti et al. (2024), STEM faculty in the SEA program are motivated to iteratively advance their pedagogy to support their students to engage productively in their classrooms. We therefore hypothesize that the current undergraduate science curriculum, which does not center authentic research, shapes both teaching and the learning experience in ways that fundamentally underpin the low persistence of undergraduate students in STEM.

## Limitations

The central limitation of the current study is that all the instructors who participated in this study came from the same program, thereby reflecting a form of selection bias. The SEA-PHAGES program has an extensive network of instructors and schools and, as an iREC that is supported by funding from the Howard Hughes Medical Institute, has extensive interaction between instructors. This networked aspect of this program may have a facilitative aspect to the construction of a particular type of professional identity. It is unclear from the present study if instructors of CREs beyond the SEA-PHAGES program have a similar construct. This will need to be addressed in future research. A second limitation of the current study relates to the way the data were collected and analyzed. The process of data collection and analysis involved multiple stages of large group discussion and explication. This process can in itself lead to a higher degree of convergence than might happen with other data elicitation methods. While this is conducive for explicating shared identities that are agreed upon, it is does not lend itself to the divergences which may be present.

## Conclusions

As exemplified in this study of professional identity, the way STEM faculty see themselves in the context of their classroom shapes their instruction and the corresponding educational experience and outcomes for their students. What this study makes clear is thus the value, if not necessity, of committing to STEM faculty in ways that affirm the type of professional identity described in this study. STEM faculty who are supported to engage in pedagogy that reflects this professional identity will afford their students rich educational experiences and outcomes. The traditional lab and lecture formats do not support this identity; the SEA iREC and the SEA-PHAGES CRE do, and serve as frameworks and examples for the types of resources and classrooms that should be afforded to all STEM faculty, as described in Hanauer, et al., 2017 and Monti et al., under review. In particular, STEM faculty should be supported to integrate the roles as a researcher and as an educator, and to operate in communities that share such an integrated identity, be that within their departments, programs, various scientific organizations, and institutions. As seen here, such an integrated professional identity is not only possible but is also a far more positive identity position for faculty to occupy.

## Figures and Tables

**Table 1:** Overview of the data collection process and resultant data

Data Collection Stage	Process Description	Resultant Data
*Reflective Journaling*	For a year, 97 CRE faculty wrote weekly reflections in a provided dedicated journal on meaningful events that happened during their week of teaching	A *reflective journal* with weekly entries and comments on notable teaching experiences with students. On average participants had 18 weeks of reflective comments on their teaching
*Autoethnographic Writing*	Following the completion of the year of reflective writing, an online Zoom writing workshop was held with 46 CRE faculty. Each instructor read through their reflective journals and chose 3 of their entries that in their opinion represented their work and interaction with their students as a CRE instructor. Following this they wrote a description of their professional identities as CRE instructors.	A series of *3 weekly journal entries* considered characteristic of the instructors work as a CRE instructor and a *written description of their professional identities*.
*Small Group Evaluation and Writing*	A second writing workshop was held in which faculty who participated in the first workshop shared their professional identity descriptions with a small group of other faculty. The CRE instructors were tasked to define their collective professional identity	A collection of written descriptions of *shared professional identity*
*Individual Identity Differentiation*	The final part of the workshop in which a shared professional identity was defined, faculty were asked to think of ways in which they differ from the shared identity. Faculty were asked to specify points at which their personal identities differed from the group identity. Following the workshop, the written descriptions of individual faculty identities were submitted.	A collection of *written descriptions of individual faculty identities* interacted with the specified shared professional identity.
*Community Checking and Validation*	Once the data had been analyzed, it was presented to over 100 faculty at an annual meeting of CRE instructors. The analyzed data for the definition of the professional identity of a CRE instructor was presented and discussed by small groups of instructors. A survey was presented to faculty to assess the quality of the data and to make revisions and modifications to the presented categories and analyses.	A survey summarizing the *degree of agreement with presented analyses* and *recommendations for modification* or revision.
*Community Explication of Inclusive CRE Instruction Vignettes*	A final meeting was held with a large group of instructors to explicate the ways in which CRE instruction may manifest aspects of inclusive educational practices. In small groups faculty were presented with specific entries from the year-long diary study chosen for their relevance. Each vignette was discussed in a small group and following the session each instructor completed a survey in which they presented their understandings.	A list of written explications of the way in which specific instructors understand how inclusive education is manifest in CRE instruction

**Table 2:** Demographic Features of Faculty Participants

		N	%
*Rank*	Full Professor	31	32.6
	Associate Professor	26	27.4
	Assistant Professor	19	20.0
	Clinical (non-tenure) Professor	1	1.1
	Instructor	12	12.6
	Graduate Teacher Assistant	1	1.1
	Other	5	5.3

*Teaching Position*	Full Time	85	88.5
	Part Time	11	11.5

*Gender Identification*	Woman	65	68.4
	Man	28	29.5
	Unlisted	2	2.1

*Tenure Status*	Tenured	57	60.0
	Not-Tenured	38	40.0

**Table 3:** Definitions of General Categories of Professional Identity and the Percentage of Overall Community Agreement

General Categories	Definition	Community Agreement %
*Values*	The ways of behaving that a person considers important in their professional life	91.43
*Roles*	The different types of professional role an instructor has with all associated expectations and meanings.	88.58
*Sense of Self*	The personal characteristics that are associated with being a CRE instructor	85.72

**Table 4:** Specification of the values of CRE instructors with a definition, example statements and percentage of community agreement

Specification	Definition	Example Statements
**CRE Inclusivity**	Behaving in a way that facilitates and supports that every student is treated fairly and equitably so that there is equal opportunity to conduct authentic research	“I conform in terms of the commitment to broadening undergraduate research education particularly among UR (underrepresented) groups who otherwise would not have access to a research experience. “
**Student Success**	The belief and set of behaviors that each student can meaningfully complete their authentic research experience successfully from a scientific, educational, and personal perspective.	“Additionally, all SEA-PHAGES faculty overwhelmingly share a passion for our students and their success, and are invested emotionally in this process. Student success, whether it be research success, content mastery, or some other desired outcome, is our success, too; we take great pride in our students and their work. I share in their joy and excitement every semester at the first appearance of plaques, encourage them when experiments fail, and admire their vast accomplishments when they present their scientific findings in posters or presentations”
**Community Membership**	The belief and set of behaviors that make every student feel that they are a member of large scientific community dedicated to the production of shared scientific knowledge	“I think providing students an opportunity to build community and connections with each other and with me in a class/lab environment that is welcoming, comfortable, collaborative rather than competitive, and open is really important, especially for first-year students – a big part of my professional identity in these courses is tied to fostering this type of environment for students”
**Ownership/Agency**	The belief and set of behaviors that see every student as an authentic researcher and learner who has control, ownership and agency over their research and education	“I think the best part of teaching is seeing that students can take what they have learned in introductory and other courses, put it together, and do it on their own in different contexts and produce a professional product.”
**Science**	The belief and set of behaviors that the authentic research conducted by students in the lab is important to a wider community of scientists, may have value for solving real-world issues and can help us to understand of the world we live in.	“SEA-PHAGES faculty often treat students as scientific colleagues during the phage discovery process. In my own experience during the bioinformatics semester, we discovered an extremely unusual pattern of infection in which one phage seemed to infect an alternative host better than the original isolation host, and this difference was striking in terms of plaque morphology and titer. I was so excited to see these results when I looked in the incubator, and it was hard for me to avoid spoiling the results for students. I wanted to make sure that students “did the driving” in terms of interpreting these results, so I needed to tone down my level of excitement at the results until the class was able to reach conclusions together.”
**Overcoming Failure**	The belief and set of behaviors that see authentic scientific research as unpredictable and involving failure and that the role of the professional scientist is to learn from this and overcome presented challenges.	“students would be forced into difficult, uncomfortable situations where the results could not be predicted, and failures could sometimes not be explained. They had to be able to show the students the educational value of failure, in that their time was not wasted by repeating experiments but instead provided with a new avenue to learn, oftentimes more than if the experiments went perfectly. “
**Persistence**	The belief and set of behaviors that support all students in continuing their educational path even though there are difficulties along the way	“I’m deeply invested in helping students transition/acclimate to college-level learning and strongly believe that incorporating both authentic experiences in biology (i.e. phages) and metacognition into first-year courses can help students from broad backgrounds/levels of preparedness move through this often-challenging time.”
**Degree of Community Support: 91.43%**		

**Table 5:** Specification of the roles of CRE instructors with a definition, example statements and percentage of community agreement

Specification	Definition	Example Statements
**Mentor**	A role in which the instructor facilitates the development of a student researcher by providing advice, emotional support, personal training and feedback.	“The paragraph describes a committed teacher and a caring mentor. A mentor who motivates students and helps them grow. Person who has working knowledge of the science necessary to conduct the project and to run safely lab full of students.”
**Advocate and Counselor**	A role in which the instructor provides support and advice that help a student to overcome the barriers that emerge as a student researcher or as a student more broadly.	“One of my discussion colleagues noticed a thread through my responses is that I act like a coach and watch students work (individual or group) and step in only when they are struggling and need support”
**Scientist**	A role in which the instructor works and shares with student researchers their research practices, findings and communities in order to facilitate the furthering of scientific knowledge.	“I am a member of a community of scientists and educators who work collaboratively to seek insights into bacteriophage evolution and enhance STEM education”
**Educator**	A role in which the instructor uses their experience and knowledge of science and pedagogy to advance each student’s knowledge, ability and disposition to complete their science education.	“We train students in a variety of laboratory and bioinformatic techniques and guide them as they analyze their results. As teachers, we teach scientific communication skills by having students document their work in a notebook and present their findings in written and oral forms”
**Motivator**	A role in which the instructor recognizes and announces the successes of students and encourages and inspires students during their research.	“I love that students think that I exude enthusiasm for the work they do in the lab and for the importance of their contributions to understanding aspects of phage biology.”
**Collaborator**	A role in which the instructor works together with students for the shared aim of creating scientifically valuable output.	“Early in the semester I am the expert, and they are the learner, however, as the semester goes along, we are more colleagues working towards a common goal”
**Community Builder**	A role in which the instructor creates for students a sense of community in the lab and with the broader community of scientists.	“I have always seen the main role of my course as building community, and my professional identity is mainly to facilitate the connections in this community”
**Learner**	A role in which the instructor learns new skills or knowledge in relation to science and educational issues	“The program provides opportunities for professional development in both scientific and education research, and therefore I have been able to continuously grow and learn in both areas.”
**Evaluator**	A role in which the instructor assesses and evaluates both formally and informally the scientific work of the student researcher	“I use weekly reflection assignments to encourage students to relate phages and bacterial hosts to the topics discussed in the lecture portion. Having to correlate topics like photosynthesis to phages and bacteria can be challenging but moves the students away from memorization and to building connections. For example, I have had students’ reflections relate photosynthesis to phages by discussing a journal article that they found about cyanobacteria phages”
**Project Manager**	A role in which the instructor oversees the development of the students’ research projects and provides organizational support for the work being conducted.	“It is critical that SEA instructors are highly organized and well prepared for lab meetings, as it would be impossible to manage the many intersecting, complex protocols without these skills. However, as things will inevitably not work perfectly the first time and plans will change, they must also be highly adaptable, and have the confidence to manage those adaptations.”
**Degree of Community Support – 88.58%**		

**Table 6:** Specification of the sense of self of CRE instructors with a definition, example statements and percentage of community agreement

Specification	Definition	Example Statements
**Dedicated**	A sense of commitment, passion and persistence to help students and the scientific community as a whole to continue their shared research.	“To belong to a network of scientists with such passion for their work and dedication to their students is invigorating and inspiring”
**Resilient and Resourceful**	A personal quality of the ability to respond in a variety of ways to novel situations and have the mental toughness to be able to contend with the unplanned outcomes and experiences of scientific research.	“My goal as a faculty is to be knowledgeable, confident in what I do and do not know, and flexible to be able to pivot or adapt to changing conditions”
**Pride in Students**	The sense of personal satisfaction and accomplishment with student scientific and educational development and successes.	“I am >30 years into my career as a University Professor. I have never been involved in a program that has given me so much joy from the student perspective. To get to watch in real time the growth and development of these young scientists is a reason I get up in the morning and still put in 60hrs a week. What I love doing in thinking up creative ways to make the challenge of research more approachable”
**Multi-Skilled**	A personal quality of being able to function in a wide range of different roles required in order to manage a course-based research lab	“All of us are a jack of all trades, meaning that we do most all of the prep, teaching, grading and engagement of the course.”
**Valued**	A sense that the educational and scientific work that the instructor performs is appreciated and respected by the students and broader scientific community.	“We are valued for what we bring to the community in both the education and scientific areas. There is shared ownership of discoveries.”
**Community Member**	The sense that one is a participating, contributing and valued member of a community of researchers.	“I strongly believe that I am functioning in an environment where I am on the same team with my students - we are in the research process together, neither of us knowing the outcome of the discoveries to unfold and all excited as we anticipate the next steps. I feel so comfortable within the community of SEA-PHAGES faculty”
**Responsible**	The personal quality in which the instructor feels accountable for the student’s research and development.	“I feel a demanding sense of responsibility for student success, desiring to keep everyone engaged in the research process, and at times I blame myself when things go wrong.”
**Overworked**	The sense that the work and responsibilities of an instructor in a CRE are extensive and can feel overwhelming.	“Stress and frustration was more apparent in earlier days in the semester, as I had to re-calibrate my expectations of students at this level and encourage them to be patient and accommodating to changes that were bound to come, and to encourage them to enjoy creativity, exploring the unknown, and to take reasonable risks in presenting their ideas about their experimental outcomes.”
**Degree of Community Support - 85.72%**		

## Appendix A

Below are the prompts for all the different stages of the investigation.

### A. Weekly Reflective Journal Prompt:

“As a first stage of this project, we would like you to keep a reflective diary of your SEA-PHAGES instruction. Please use the provided journal to make your entries. In the later stages of this project, you will be returning to use these diary entries so please make sure that you can find them at a later date. In the diary we would like you to make the following two entries on a weekly basis:

1. **Noticeable Moments**: Please briefly describe any moment during your course time which caught your attention. A noticeable moment could be a comment made by a student, an event that happened in class, an unexpected outcome…etc. Basically, anything that surprised you or caught your attention during this week’s work. Your description can be in note form but please provide enough detail so that you can remember what was involved in the specific event you are describing.
2. **Reflective Comments**: Please briefly reflect on the week that has passed. Consider your educational and research activities over the last week. What characterized your work in the SEA-PHAGES program this week? Do you have any observations or comments about your teaching or research in the SEA-PHAGES program? Please briefly in writing reflect on the past week in the SEA-PHAGES program.

Your weekly entries in the reflective journal will not be monitored or collected by the program. The diary is completely your own and you will have at all times have control over what you decide to share or not share with other members of this project. As such, please include all your honest self-reflections, comments or events which might occur. The role of the reflective diary is to help you with the next stage of this project in which you will write an integrative summary with a writing workshop setting.”

### B. Autoethnographic Writing Workshop Prompts

“Individual Work: As a first stage in this writing workshop, I would like you to think carefully about your experiences as a SEA-PHAGES instructor over the last year. Please do not think in “general” terms about the experiences. But rather, I would like you to flip through your journal and choose three entries from your reflective teaching journal that either stand out to you as significant or are what you consider to representative of your work as a SEA-PHAGES instructor. Please note the page (as you go through the journal) and also make a list on a separate document by giving this experience/journal entry a title.

Please look at your list of 3 experiences. For each experience, close your eyes for a moment and try to relive the experience as much as is possible. Once you have done this write out as much as you can in describing the experience. Address your feelings, thoughts and what you can see and did during this event. Don’t worry about grammar and spelling. Just write continually for 5 minutes for each experience.

At the end of this process, you should have notes on three experiences that you consider either significant or representative of your work as a SEA-PHAGES instructor

Pair work: Introduce yourself to your partner. This part of the process needs to be done in two stages so please be cognizant of the time. The first partner should present their first chosen experience. Please describe it to your partner in as much detail as you can. The second partner should listen and ask any clarification questions that are necessary for you to get a good understanding of what is being described. As you listen please consider how you would have behaved, felt, responded, acted….etc. Please express any thoughts you have in relation to the experience being described. The role of the listener is to offer additional ways of seeing the professional experience being described. Comment on anything that seems interesting, strange worthy of comment …etc. Go through all three experiences one after the other. Please write out notes on each of the experiences, especially any observations that are beyond your own initial thoughts, Then, please move to the second partner and go through all three experiences

Autoethnographic Writing: After this session, please write out a full detailed description of each of the three experiences you have chosen to present. When you write out the description make sure that you provide full details about the experience including your feelings, thoughts, comments, and interactions. Please include any ideas and thoughts from your interaction with your partner. Any new ways of seeing this event and understanding your way of being are very important.

Please submit your 3 descriptions to the survey that will be sent to your email.”

### C. Small Group Evaluation and Writing Prompts

“As a group, answer the following question: What is it that we share with other SEA instructors? List all the behaviors, knowledge, values, judgments and persona that you share with other faculty members. Try to get at what is the shared aspect of being a SEA-PHAGES instructor. Please create this list and description together as a group. Post your list and description to the link provided.”

### D. Individual Identity Differentiation Writing Prompts

“Please look at the shared document you have just written and consider the points at which you diverge from this description. As an individual, answer the following question: In what ways do you diverge and conform to the description of the shared SEA-PHAGES faculty identity? Describe your professional identity and sense of belonging, acceptance, agency, identification beliefs and values. Please provide examples from your own experience of what it is like for you to be a SEA community member and instructor. Please write out your description of your professional identity on a word document and then upload it (or post it) into the link that will be provided for you”

### E. Community Checking and Validation Prompts following Presented Analysis

*“*Do the VALUES, ROLES and SENSE of SELF specified in this analysis of shared professional identity make sense to you? Are there any VALUES, ROLES and SENSE of SELF that you consider part of your professional identity as a CRE instructor that should be added to this list? Are there any VALUES, ROLES and SENSE of SELF that you consider part of your professional identity as a CRE instructor that should be deleted from this list? What are your thoughts on the full model of professional identity? What would you like to add to this model? Any there any components that you think should be deleted from this model?”

### F. Community Explication of the Manifestation of Inclusive Education in CRE Instruction

“Read each of the vignettes and discuss it together in terms of what features of shared professional identity come into play and how CRE instruction facilitates inclusive education. What is the instructor doing that facilitates an inclusive educational experience? What values, roles and sense of self come into play in this vignette?”
